# Supplementary material for: THOI: An efficient and accessible library for computing higher-order interactions enhanced by batch-processing
Source: PLoS One. 2026 May 11;21(5):e0348005. doi: 10.1371/journal.pone.0348005 (PMC13160338; doi:10.1371/journal.pone.0348005)
Supplement: S1 Text — (PDF) [file pone.0348005.s001.pdf]

Supporting information

Mathematical basis

Key concepts HOI hold the potential to uncover intricate statistical relationships in complex systems by offering a principled way to partition the system information. Here, we used and information theoretic approach to HOI, where concepts such as entropy, mutual information, Synergy, and Redundancy are fundamentals.

Entropy Entropy is a measure of the unpredictability or randomness of a system. In information theory, it represents the average amount of information or surprise produced by a stochastic source of data. Higher entropy indicates more uncertainty or complexity within the system.

Mutual information Mutual information (MI) is a measure of the amount of information that two random variables share. It quantifies the reduction in uncertainty about one variable given knowledge of another.

Synergy Information that can only be accessed when observing the system as a whole. Occurs when the combined information from multiple variables provides more information than the sum of their individual contributions.

Redundancy Information that is present in multiple variables. Occurs when copies of the same information can be retrieved from different parts of the system.

Entropy analytical expression for multivariate Gaussian variables The entropy formula requires knowledge of the probability density function $p(X^n)$. In most cases, this function is unknown. However, a notable exception is when the data follows a normal distribution, for which the entropy has a well-established, closed-form analytical expression:

$$H(X^n) = \frac{1}{2}\log((2\pi e)^n|\Sigma|) \tag{14}$$

Where Σ is the covariance matrix and $|\Sigma|$ its determinant.

As $H(X^n)$ depends only on the covariance matrix Σ, the problem of entropy estimation for multivariate Gaussian variables is reduced to the estimation of its covariance matrix. However, due to finite sample effects, this estimation is biased. Then, for a multivariate system of n variables and T samples, the bias corrector for entropy estimator follows [S1]:

$$\eta(x) = \frac{1}{2}n\ \log\left(\frac{2}{T-1}\right) + \sum_{j}^{n}\Psi\left(\frac{T-j}{2}\right) \tag{15}$$

where Ψ is the digamma function. This bias is subtracted from the estimated entropy when dealing with experimental data.

Estimation of entropy via Gaussian copulas

Current research has explored the estimation of multivariate mutual information using Gaussian copulas, which provide a flexible and robust approach to capture

dependencies between random variables [30, 33]. Gaussian copulas allow for the separation of marginal distributions from the joint dependency structure, enabling more accurate entropy estimation, particularly when the underlying distributions deviate from normality [S2].

As delineated in equation 14, the Gaussian formula provides a robust framework for systems that adhere to a normal distribution. However, real-world data often deviate from this idealized distribution. In instances where the distribution of the system is not strictly normal but remains approximately normal, Gaussian copulas offer a powerful alternative This approach have been used specially in neuroscience both for bivariate and multivariate problems.

Copulas Formally, a n-dimensional copula is the cumulative distribution function (CDF) $C\left(u_1, \ldots, u_n\right): [0,1]^n \rightarrow [0,1]$ of a vector of random variables defined on $[0,1]^n$ with uniformly distributed marginals $\mathcal{U}_{[0,1]}$ over [0,1]

$$C\left(u_1, \ldots, u_n\right)=P\left(U_1 \leq u_1, \ldots, U_n \leq u_n\right) \tag{16}$$

where $U_i \sim \mathcal{U}_{[0,1]}$

The Sklar's theorem states that any multivariate distribution can be described by separately specifying the marginal distributions and the copula [S3]. Formally, states that for a n-dimensional random vector $X=(X_1, \ldots, X_n)$, let F_X be its CDF with marginals CDFs $F_i(x)=P(X_i \leq x)$ and a copula function $C:[0,1]^n \rightarrow [0,1]$, such that $\forall x \in \mathbb{R}^n$:

$$F(x_1, \ldots, x_l)=C(F_1(x_1), \ldots, F_k(x_l)) \tag{17}$$

If the marginals F_i are continuous then the copula C is unique. Therefore, a joint distribution F_X can be split into marginals and a copula. Conversely, if C is a copula and $F_1, \ldots, F_n$ are CDFs, then function $F_X=C\left(F_1\left(x_1\right), \ldots, F_n\left(x_n\right)\right)$ is a n dimensional CDF with marginals $F_1, \ldots, F_n$. Sklar's theorem relates the copula to the joint distribution function of the variables $U_i=F_i\left(X_i\right)$.

$$C\left(u_1, \ldots, u_n\right)=F_X\left(F_1^{-1}\left(u_1\right), \ldots, F_n^{-1}\left(u_n\right)\right), \quad u_i \in[0,1] \tag{18}$$

where F_i^{-1} are the inverse CDFs. For a differentiable copula C, we can define the *copula density function* as

$$c\left(u_1, \ldots, u_n\right)=\frac{\partial^n}{\partial u_1 \ldots \partial u_n} C\left(u_1, \ldots, u_n\right). \tag{19}$$

Consider $u_i:=F_i\left(x_i\right)$ the cumulative distribution functions and f_i its corresponding PDFs corresponding to the CDFs $F_i(\cdot,$. The *copula density* function can be written as

$$c\left(u_1, \ldots, u_n\right)=\frac{f_X\left(F_1^{-1}\left(u_1\right), \ldots, F_d^{-1}\left(u_n\right)\right)}{\prod_{i=1}^n f_i\left(F_i^{-1}\left(u_i\right)\right)} \tag{20}$$

Gaussian copulas Now let $X \sim \mathrm{N}_n(\mathbf{0}, \Sigma)$, where Σ is the covariance matrix of X. Then the corresponding Gaussian copula is defined as

$$C_P^{Gauss}(\mathbf{u}) := \Phi_\Sigma\left(\Phi^{-1}(u_1), \ldots, \Phi^{-1}(u_n)\right) \tag{21}$$

where $\Phi(\cdot)$ is the standard univariate normal CDF and $\Phi_\Sigma(\cdot)$ denotes the joint CDF of X.

Using a Gaussian copula, we transform the marginal of the dataset to a uniform scale and subsequently apply the inverse of the standard normal cumulative distribution function to achieve normally distributed marginals. Then, the covariance matrix of the Gaussian copula transformed can be easily estimated.

Specifically, for data with T samples, the Gaussian copula is can be easily implemented following:

$$\Sigma_{GC}(X) = \Sigma\left[\Phi\left(\frac{rank(rank(X))}{T+1}\right)\right] \tag{22}$$

where Σ is the covariance operator that takes a $n-$variate system X with shape (n, T) and computes a covariance matrix of shape (n, n). Φ is the inverse CDF of the standard normal distribution and $rank(\cdot)$ is the ranking function.

Once we obtain this covariance matrix, we can then calculate the Gaussian entropy following equation 14, which serves as an lower bound for entropy in data that is not normally distributed. This approach leverages the closed form of entropy for Gaussian variables, avoiding costly non parametric estimations of probability density functions by reducing the problem to the estimation of the covariance matrix of the copula-transformed data, which requires much less samples.

The combinatorial explosion

To properly capture the information structure within a system, especially when using O-information, we should consider all possible groupings or "n-plets" of components (i.e. combinations of n variables), from triplets up to the full set of components involved. For a system with n variables, the number of possible combinations increases exponentially with n. The number of ways to choose k components from n is given by the binomial coefficient $\binom{n}{k}$. From the binomial theorem, we know the following:

$$\begin{aligned} \sum_{k=0}^{n}\binom{n}{k} &= 2^n \\ \binom{n}{0}+\binom{n}{1}+\binom{n}{2}+\sum_{k=3}^{n}\binom{n}{k} &= 2^n \\ \sum_{k=3}^{n}\binom{n}{k} &= 2^n - 1 - n - \frac{n(n-1)}{2} \end{aligned} \tag{23}$$

Computing O-information for each combination involves evaluating the joint entropy of variable subsets, requiring significant data manipulation and computational resources. Since joint entropy must be calculated for every possible subset, this exhaustive approach ensures no potential interactions are overlooked, but it imposes a high computational burden. The complexity escalates as the number of variables increases, often making the full computation infeasible. This challenge is especially relevant in fields like neuroscience, where systems may consist of hundreds or thousands of units, such as neurons or brain regions

Probabilistic graphical models (PGM)

To generate data where the ground truth value of the Ω is known, we use probabilistic graphical models (PGM) as introduced in [S4]. PGMs are structured representations of the conditional dependencies between random variables in a system. It uses a graph where nodes represent the variables, and edges represent the probabilistic relationships between them. These models have been used to generate synergistic (Head-to-Head) and redundant (Tail-to-Tail) systems, regardless of the probability distribution [19]. Here we add a parameter c that controls the level of synergy or redundancy on each system, respectively.

Synergy system (S-system): In a S-system configuration, each variable $X_1, X_2, \ldots, X_n$ is marginally independent, and a variable Y depends on all of them. In this model, arrows in the graphical model point from each X_i to Y, meaning that Y is influenced by the joint state of the X_i's weighted by the parameter c. Mathematically, this model is expressed as:

$$p(X^n, Y) = p(Y \mid X^n, c) \prod_{j=1}^{n} p(X_j) \tag{24}$$

In this case, the variables $X_1, X_2, \ldots, X_n$ are independent, but their collective interaction determines Y, leading to synergistic interaction between any group of variables comprising Y and at least two different X_i variables.

If all variables are Normally distributed, the covariance matrix, Σ_S, of the S-system follows:

$$\Sigma_S = \begin{bmatrix} \mathbf{I}_n & c\mathbf{1}_n \\ c\mathbf{1}_n^\top & nc^2 + 1 \end{bmatrix} \tag{25}$$

where $\mathbf{I}_n$ is the $n \times n$ identity matrix and $\mathbf{1}_n$ is an $n \times 1$ vector of ones.

Redundancy system (R-system): In an R-system configuration, variables $X_1, X_2, \ldots, X_n$ are conditionally independent given Y. In this model, arrows in the graphical model point from Y to each X_i, meaning that Y acts as a common source weighted by c for all X_i's. Mathematically, this model is expressed as:

$$p(X^n, Y) = p(Y) \prod_{j=1}^{n} p(X_j \mid Y, c) \tag{26}$$

This structure suggests that all dependencies among the X_i variables are explained through Y, leading to redundant information across the variables because they all depend on the same source.

If all variables are Normally distributed, the covariance matrix, Σ_R, of the R-system follows:

$$\Sigma_R = \begin{bmatrix} c^2+1 & c^2 & \cdots & c^2 & c \\ c^2 & c^2+1 & \cdots & c^2 & c \\ \vdots & \vdots & \ddots & \vdots & \vdots \\ c^2 & c^2 & \cdots & c^2+1 & c \\ c & c & \cdots & c & 1 \end{bmatrix} \tag{27}$$

Independent (I): In a **Independent** configuration, variables $X_1, X_2, \ldots, X_n$ are independent variables with some given probabilistic distribution. No statistical interdependence is present.

Concatenated system: This system consists in concatenating different systems without interactions between them, being each system either a R-system, a S-system (with some modulation of the c parameter) and a set of independent variables. Specifically, we created a concatenated system composed of 5 sub-systems. Two redundant (one weak with $c = 0.5$ and one strong with $c = 1$), two synergistic (one weak with $c = 0.5$ and one strong with $c = 1$) and one independent. Each system was composed of 20 variables, creating a system of 100 variables in total. Because Ω satisfies the additive property for independent components ($A \perp B \Rightarrow \Omega(A + B) = \Omega(A) + \Omega(B)$), we can know the Ω for the whole system. Using the aforementioned ground-truth covariance matrices, the entropy equation (equation 14) and THOI, we computes all the ground truth metrics of each system.

Number of repeats in Greedy algorithm

To complement the analysis presented in the main text, we examine how the performance of the greedy algorithm varies with the number of random initializations in a system of $N = 30$ variables. As shown in Supporting Information Fig 7, the algorithm converges more quickly to the global optimum in the redundancy (maximization) case than in the synergy (minimization) case. In the synergistic scenario, the number of variables selected by the GA decreases from 19 (initially including all strong and weak synergistic variables along with 7 unrelated ones) when using only 2 repeats, to the optimal 12-variable subset (comprising only the strong and weak synergistic variables) with 2000 repeats. This convergence follows a roughly logarithmic trend, with approximately two incorrect variables replaced per order-of-magnitude increase in the number of repeats.

Fig 7. Dependence of the greedy algorithm with the number of repeats. Same as Fig 4A, C and E, but for a system with N=30 variables. Left panel shows the maximum (red) and minimum (blue) O-information for different number of repeats (initial conditions). Center and right panel shows the variables involved in the optimal solution for maximization and for minimization, respectively, using 2000 repeats (the optimum).

Stability of simulated annealing across orders of interaction

As SA algorithm is stochastic, we assessed the convergence of the solutions of the SA algorithm across different initial conditions. Fig 8 reports the stability of the SA heuristic under Ω minimization and maximization across increasing orders of interactions for the same synthetic systems used in 4 . We extracted the distribution of optimized Ω values and the selection frequency of variables by interaction type. Across all orders of interactions, the SA converges to stable solutions with sharply peaked Ω distributions and selection patterns that match the ground truth. Synergistic-dominated solutions dominate under Ω minimization, redundancy-dominated solutions dominate under Ω maximization, and independent variables are incorporated after including variables of some of the other systems.

Order-free In the order-free optimization setting, Ω distributions are extremely narrow, indicating strong convergence (minimization: $\Omega = -6.3373 \pm 0.00237$;

Fig 8. Stability of the simulated-annealing optimization heuristic applied to a 100-variable system composed of five concatenated subsystems: weak redundancy (WR), strong redundancy (SR), weak synergy (WS), strong synergy (SS), and independence (I). Each subsystem contains 20 variables (see supporting information Concatenated system). Rows correspond to increasing order of interaction at which the algorithm is evaluated (order-free, 20, 40, 60, and 80), while columns report results obtained under Ω minimization (left) and Ω maximization (right). For each condition, the upper panel shows the distribution of the obtained Ω values across repeated optimization runs (histograms expressed as the percentage of runs per bin), whereas the lower panel shows the selection frequency (%) of variables across runs, grouped by subsystems. Selection frequency quantifies how often variables belonging to each subsystem are included in the optimal solution. Colors are consistent across all panels and denote different subsystems.

maximization: $\Omega = 6.3399 \pm 0.00320$). Under minimization, all variables from the weak and S-systems (WS, SS, respectively) are consistently selected, while independent variables appear with intermediate probability. Redundant subsystems are suppressed by systematically excluding the single redundancy-driving variable that induces shared information within each block (see Redundancy system (R-system)). Under maximization, the pattern is inverted, with variables from the weak and strong R-systems (WR, SR, respectively) consistently selecting variables from the S-systems only partially included without the single synergy-driving variable that induces shared information within each block (see Synergy system (S-system)).

Order 20 At order 20, the algorithm predominantly selects the SS subsystem. Under Ω minimization, the distribution centers on the negative Ω value associated with the SS subsystem ($\Omega = -1.0292 \pm 0.3471$). This reflects the predominant selection of the SS variables across runs, with occasional selection of the WS subsystem, while variables from the R-systems and independent variables are rarely included. Under Ω maximization, the distribution shifts to positive values characteristic of the SR subsystem ($\Omega = 4.7946 \pm 0.12165$), driven by its consistent selection across runs and the systematic exclusion of synergistic variables that would reduce Ω. When variables from other components appear, they systematically exclude the single variable that drives shared information as explained in the order free analysis.

Order 40 At order 40, two subsystems are selected. Under Ω minimization, the algorithm consistently recovers the SS subsystem in 100% of runs, together with the WS subsystems, yielding strongly negative and stable values ($\Omega = -5.4582 \pm 0.0826$). Independent variables are only occasionally selected, and when variables from other subsystems appear, they systematically exclude the single variable that drives shared information. This indicates that, although selection outside the synergy-dominated subsystems is possible, the algorithm preserves the interaction structure responsible for minimizing Ω.

Under Ω maximization, an analogous pattern is observed. The SR subsystem is selected in all runs, together with the WR component, producing strongly positive values with low variability ($\Omega = 6.0837 \pm 0.05406$). Independent variables and variables from non-optimal components are selected only sporadically and only when they do not include the single variable responsible for inducing synergy or redundancy dominated interactions. Overall, these results show that, even when secondary variables are occasionally included, the algorithm reliably identifies and preserves the dominant interaction structure.

Order 60 At order 60, the two most favorable subsystems are consistently included (WS and SS for minimization, WR and SR for maximization). In contrast to order 40—where selecting exactly two components is sufficient—the requirement to include an additional 20 variables forces the algorithm to incorporate variables from other subsystems. As a result, these non-optimal variables show a higher probability of being selected than at order 40, reflecting a structural constraint imposed by the order of interactions. Despite this constraint, the Ω distributions remain sharply peaked, as the heuristic preferentially selects, among the available independent variables, those that by chance still improve the solution, leading to values even better than the optimum already achieved at order 40 (minimization: $\Omega = -6.3359 \pm 0.00282$; maximization: $\Omega = 6.3408 \pm 0.00315$).

Order 80 Under Ω minimization, the SR subsystem is systematically avoided, resulting in slightly less negative but stable values ($\Omega = -5.6132 \pm 0.00283$). In this regime, the algorithm preferentially incorporates variables from the independent component while avoiding variables from the SR subsystem, even though the single redundancy-driving variable is never selected at any order.

In contrast to minimization, under Ω maximization, the additional variables required to reach order 80 are selected with approximately uniform probability from all remaining components, with the sole exception that the single synergy-driving variable of each synergistic subsystem is systematically excluded, yielding strongly positive and highly stable values ($\Omega = 6.3403 \pm 0.00315$).

Interestingly, the order-free solution closely mirrors the behavior observed at order 60. In both cases, the heuristic consistently includes the two interaction subsystems that are optimal for the objective (WS and SS under minimization, WR and SR under maximization) and fills the remaining degrees of freedom by incorporating independent variables that, by chance, improves the optimized Ω value. In the particular case of minimization, increasing the order further to 80 leads to a partial degradation of the minimization optimum, and the order-free setting correctly recovers Ω values comparable to those at order 60. This highlights the ability of the order-free formulation to identify a global optimum unconstrained by order of interactions, effectively balancing optimal component selection with neutral inclusion of independent variables.

References for Supporting Information

S1. Schürmann T. Bias analysis in entropy estimation. Journal of Physics A: Mathematical and General. 2004;37(27):L295. doi:10.1088/0305-4470/37/27/L02.

S2. Joe H. Dependence modeling with copulas. vancouver, Canada: CRC press; 2014.

S3. A S. Fonctions de répartition à *n* dimensions et leurs marges. Publications de l'Institut de Statistique de l'Universit´e de Paris. 1959;8:229-31.

S4. Koller D, Friedman N. Probabilistic graphical models: principles and techniques. Cambridge, Massachusetts: MIT press; 2009.

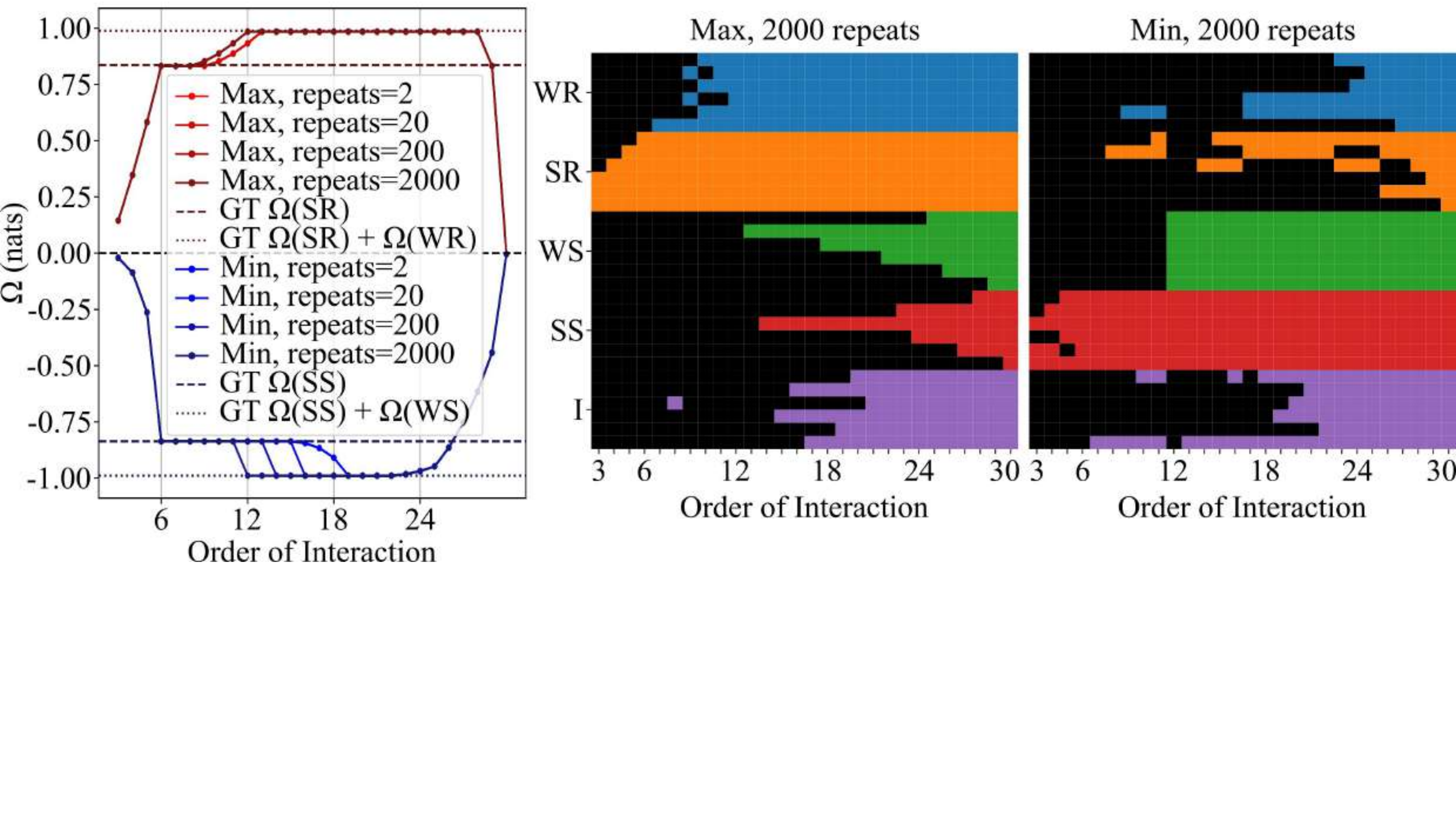

Max, repeats=2
Max, repeats=20
Max, repeats=200
Max, repeats=2000
GT Ω(SR)
GT Ω(SR) + Ω(WR)
Min, repeats=2
Min, repeats=20
Min, repeats=200
Min, repeats=2000
GT Ω(SS)
GT Ω(SS) + Ω(WS)
Ω (nats)
1.00
0.75
0.50
0.25
0.00
-0.25
-0.50
-0.75
-1.00
6
12
18
24
Order of Interaction
Max, 2000 repeats
Min, 2000 repeats
WR
SR
WS
SS
I
3
6
12
18
24
30
Order of Interaction

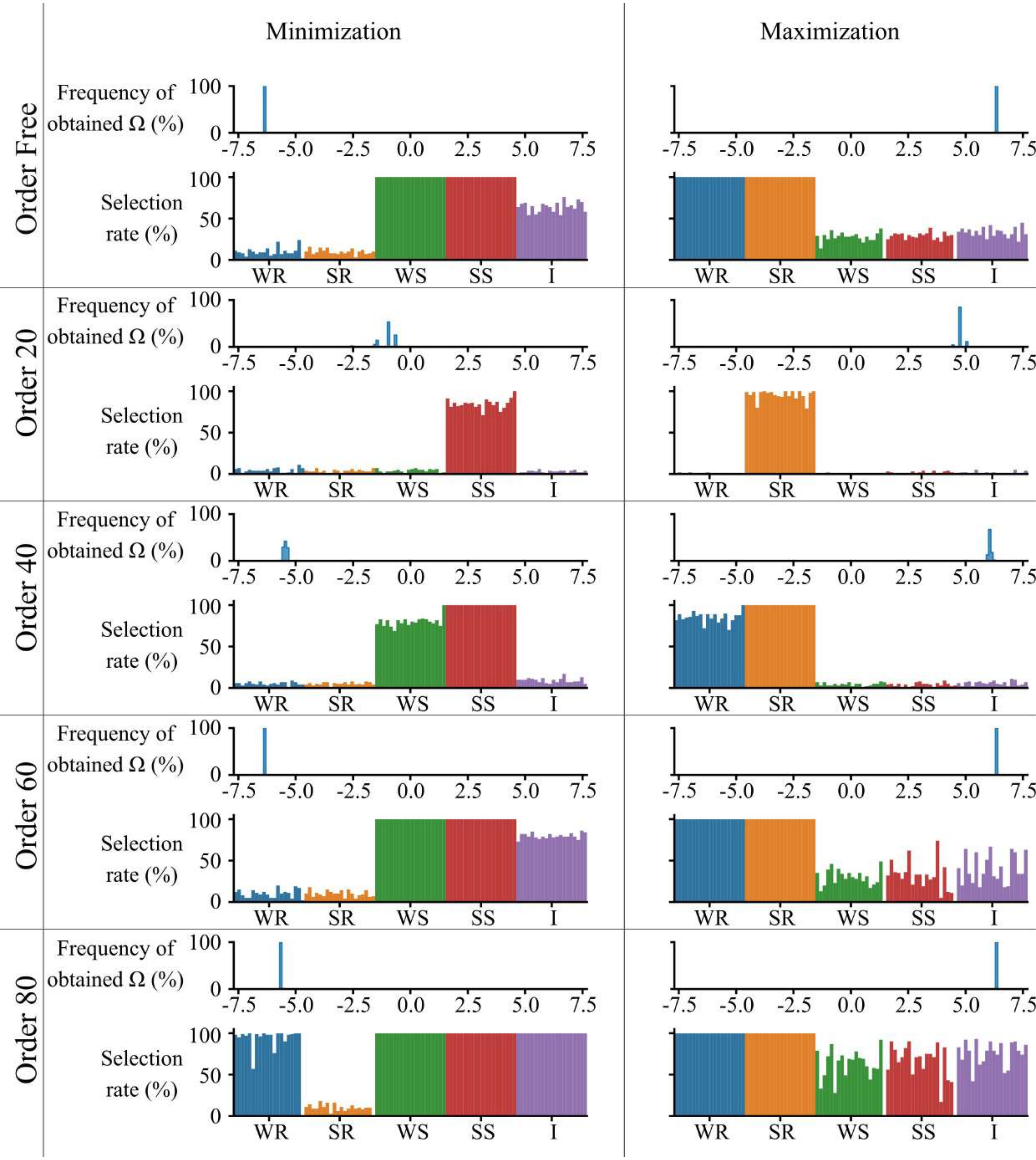

Minimization
Maximization
Order Free
Order 20
Order 40
Order 60
Order 80
Frequency of obtained Ω (%)
Selection rate (%)
100
50
0
-7.5
-5.0
-2.5
0.0
2.5
5.0
7.5
WR
SR
WS
SS
I
