# Supplementary material for: THOI: An efficient and accessible library for computing higher-order interactions enhanced by batch-processing
Source: PLoS One. 2026 May 11;21(5):e0348005. doi: 10.1371/journal.pone.0348005 (PMC13160338; doi:10.1371/journal.pone.0348005)
Supplement: Attachment — Submitted filename: response_to_reviewers.pdf [file pone.0348005.s002.pdf]

Revised manuscript submission – PONE-D-25-41547

Summary to editor

Dear Dr. Iqbal,

We would like to thank you, the Academic Editor, and the reviewers for the time and effort devoted to the evaluation of our manuscript, *"THOI: An efficient and accessible library for computing higher-order interactions enhanced by batch-processing"* (PONE-D-25-41547). We are grateful for the constructive feedback and for the opportunity to submit a revised version of the manuscript.

In response to the reviewers' comments and to the journal's editorial requirements, we have carefully revised the manuscript and fully addressed all points raised. Detailed, point-by-point responses to the reviewers and the Academic Editor are provided in the accompanying "Response to Reviewers" document, and all modifications are highlighted in the reviewed version of the manuscript as requested.

In addition, we made several structural and organizational changes to use the official LaTex template of PLOS ONE and ensure full compliance with the PLOS ONE formatting guidelines, while preserving the scientific content and conclusions of the work without any modification. We did the following structure changes:

- The original Figure 1 was reorganized to better align with the Methods–Results structure recommended by PLOS ONE. The panel describing the computational architecture is now presented as a standalone figure at the beginning of the Methods section, while the remaining panels illustrating results are presented separately within the Results section.
- The subsection previously titled *"Leveraging Gaussian entropy estimators with batch processing"*, which was originally located in the Results, has been moved into the Methods section under the broader framework describing the computational architecture.
- Several Method subsections were retitled to reflect this reorganization and to improve clarity with the revised figure placement. Importantly, these changes were purely structural and terminological; the underlying methodological descriptions, analyses and body texts remain unchanged unless highlighted as requested to facilitate the revision process.
- Following the PLOS ONE LaTeX template requirements, we removed the "Author Summary" section.
- Following the PLOS ONE Standard we renamed the following sections:
    - "Results" now is called "Results and discussion"
    - "Discussion" now is called "Conclusions"
- All figure texts are now formatted with Time New Roman to match the rest of the text

As requested by the journal requirements, we carefully reviewed the full reference list. No retracted articles were identified. During this review, we updated several citations that were previously available as preprints and have since been published in peer-reviewed journals, and we revised all references to ensure full compliance with the Vancouver citation style and PLOS ONE formatting standards.

With respect to the Data Availability Statement, we confirm that all data required to replicate the results reported in this study are publicly available through their original third-party repositories, as cited in the manuscript (OpenNeuro and Zenodo). In accordance with PLOS ONE policy, these original repositories constitute the authoritative sources for the data used in our analyses. For clarity, while our Code Availability resources include optional bundled copies of these publicly available datasets to facilitate streamlined, “plug-and-play” reproducibility, the Data Availability Statement intentionally refers only to the original public releases.

We believe that these revisions improve the clarity, structure, and readability of the manuscript, and we hope that the revised version now fully meets PLOS ONE’s publication criteria. We sincerely appreciate the positive and supportive evaluations from the reviewers and thank you again for your consideration.

We look forward to your response.

Kind regards,

Laouen Belloli

(on behalf of all authors)

Response to reviewers

Below we provide a point by point response to the issues raised by the reviewers. Our responses are written in blue

Reviewer #1: I found the present manuscript quite interesting, since it provides a very powerful tool to compute high-order interactions measures in complex systems, including the brain. The structure of paper is appropriate and figures are of great quality. Improvement in the performance of the proposed python library compared with other toolboxes in the literature is also very high. Therefore I recommend publication of the present manuscript

We thank the reviewer for his positive comments and his support for publication.

Reviewer #2: In this paper, Belloli et al. introduce a new toolbox for the efficient computation of higher-order information-theoretic measures. They not only evaluate its performance on simulated data, but also test it on multiple datasets, including human fMRI. This work represents a valuable contribution to the field, and I am highly supportive of its publication. I only have a few minor comments and questions that the authors may wish to address to improve the clarity of the narrative and the robustness of the paper.

We appreciate the positive comments and below we provide responses for the 4 points raised.

1. Figure 2 (panels C–F) could be clearer. It took me some time to understand what these panels show, and I am still not entirely certain I interpreted them correctly. Does each line correspond to one of the 20 variables in the ground truth? For example, in panel E, does an interaction order of 40 mean that all blue variables contribute to minimizing \omega, while at an interaction order of 80 the variables shown in light red also contribute?

We appreciate the reviewer's comment which helped us to improve the clarity of the figure. Indeed, the yellow vertical dashed lines indicate the order of interaction of the ground truth subsystems and their concatenation (multiples of 20). The C-F plots show on each row the 100 variables of the whole system, the order of interaction in columns, and colors denote different subsystems. If a cell is white, it means that a given variable at a given order of interaction does not contribute to the maxima/minima. To help make the figure clearer, we have added pie charts to summarize which systems were detected at each important order or interaction (20,40,60 and 80), so now we have the full microscopic detail of the variables (rows in the heatmaps) and a macroscopic description (pie charts), indicating only the subsystems. We have accordingly updated the figure caption.

2. One aspect the authors could consider testing is the stability of these algorithms. If the algorithms were run multiple times, would they consistently identify the same subset that minimizes/maximizes \omega for a given interaction order?

We thank the reviewer for raising this important point regarding algorithmic stability.

First, we clarify that the greedy algorithm used in this work is *fully deterministic*. Given a fixed starting n-plet, repeated runs always return identical trajectories, optimized Ω values, and selected subsets. Variability in the greedy results can therefore only arise from the choice of the initial n-plet, not from stochasticity in the algorithm itself. This dependence was already explicitly addressed in the original version of the manuscript by performing 100,000 repetitions starting from different triplets, thereby demonstrating convergence toward the same optimal solutions across a large set of initial conditions.

In contrast, the simulated annealing heuristic is intrinsically stochastic, motivating an explicit stability analysis as suggested by the reviewer. To address this concern, we have added a new Supporting Information section entitled "Stability of simulated annealing across interaction orders", together with a new figure (Fig. 8). We have also included a mention to this results at the end of the ' Optimization for larger systems: greedy and simulated annealing algorithms':

"Furthermore, as the SA algorithm is stochastic in nature, we tested the stability of its solutions when different initial conditions were used. We found that the solutions found by the two versions of the SA algorithm (i.e. order-free and order-specific) were highly stable in terms of the values and the variables selected in the optimal solution. See Supporting Information Stability of simulated annealing across orders of interaction for an exhaustive description."

In this analysis, we ran the simulated annealing algorithm on the same synthetic system already used in the original results. For each interaction order (order-free, 20, 40, 60, and 80) and for both Ω minimization and maximization, we performed 100 independent optimization runs, each with different initial conditions. Each run itself consisted of 100 annealing repeats, from which we retained the optimal Ω value and the associated selected n-plet, exactly as in the main manuscript. This procedure yields, for each condition, a distribution of 100 optimized Ω values and corresponding selection frequencies.

The results show that, across all orders of interactions, the simulated annealing heuristic converges robustly to stable solutions, as evidenced by sharply peaked Ω distributions with low variance and highly consistent selection patterns aligned with the ground truth. While higher orders of interactions impose structural constraints that force the inclusion of additional variables, the dominant interaction components (synergistic under minimization, redundant under maximization) are consistently recovered across runs. These findings demonstrate that the stochasticity of the simulated annealing heuristic does not compromise its stability or interpretability, and that repeated runs reliably identify the same optimal interaction structures.

3. In the analysis of the fMRI dataset, the authors could consider adding two panels to Figure 3 to show on the cortical surface which n-plets (brain regions) drive the statistically significant increases and decreases in \omega.

We agree with the reviewer that using cortical surfaces would improve the visualization of the brain areas involved. Unfortunately, the regional time series that we used were parcellated using

a custom clustering algorithm in the original publication ("*An implementation of integrated information theory in resting-state fMRI"* by Nemirovsky et al. 2023, Communications Biology). Although the publication is accompanied by the timeseries, the specific spatial location and extent of each brain region were not published with the manuscript. They provide images of the location of the used networks and respective brain regions, but not the specific locations. Reconstructing these mappings would require reprocessing the full raw fMRI dataset and replicating the original clustering procedure, which is beyond the scope of this methods-focused contribution. We therefore retain the fMRI analysis as a proof of concept illustrating the applicability of THOI, and we now explicitly note this limitation in the manuscript.

4. In the analysis of large databases of synthetic and real-world systems, the authors report correlations between features of the information-theoretic measures. How do the authors interpret the finding that several features (e.g., pro syn n-plets, order min \omega, and min \omega) are strongly anticorrelated across datasets?

We appreciate the reviewers' comments on this. Indeed, the anti-correlations are interesting as they express the different qualities of interdependence that these measures capture. The proportion of synergistic n-plets, the order of min Ω and the min Ω are the only features with the negative loading in the 1st principal component, mirroring their negative values in the correlation matrix. This indicates that these features characterize systems in which multivariate dependencies are not driven by strong low-order interactions. Strong interdependencies at lower orders (encoded by pairwise mutual information) lead to redundancy at higher orders, which is also demonstrated by the positive correlation between the mutual information features and the max/mean/whole TC, DTC, Ω and S. In these regimes, higher-order interactions largely inherit structure from lower orders. Furthermore, this is also consistent with the results of the Fig 4 (optimization), where we see that the max Ω grows linearly with the order of interactions, while synergy grows nonlinearly, indicating that strong dependence at lower order leads to redundancy but not to synergy.
In summary, these 3 features are encoding the numerosity and depth of synergy-dominated n-plets across orders, capturing a regime that is orthogonal to low-order, redundancy-dominated structure, represented in the 3rd principal component. We have now included this interpretation in the 'Analysis of large database of synthetic and real-world systems' Results subsection:

"*The negative loadings correspond to synergy-related features (proportion of synergistic n-plets, order of min Ω, and min Ω), which mirror their negative values in the correlation matrix (Fig 6A). This indicates that strong low-order interdependence is associated with increasing redundancy at higher orders, whereas synergy-dominated interactions preferentially arise when low-order dependencies are weaker".*

[...]

"This component captures a regime complementary to Overall Interdependence, characterized by weak low-order interactions and a high numerosity of synergy-dominated n-plets. It reflects both the prevalence of synergistic interactions and their depth across orders. Together with the first component, this component highlights that synergy is not driven by strong pairwise dependence, but is instead predominantly realized at higher orders."
